# Twitter Mediated Sociopolitical Communication During the COVID-19 Pandemic Crisis in India

**DOI:** 10.3389/fpsyg.2021.784907

**Published:** 2021-12-24

**Authors:** Nishtha Jain, Preet Malviya, Purnima Singh, Sumitava Mukherjee

**Affiliations:** ^1^Department of Humanities and Social Sciences, Indian Institute of Technology Delhi, New Delhi, India; ^2^Department of Electrical Engineering, Indian Institute of Technology Delhi, New Delhi, India

**Keywords:** crisis communication, COVID-19, political leaders, India, Twitter, social media

## Abstract

While Twitter has grown popular among political leaders as a means of computer-mediated mass media communication alternative, the COVID-19 pandemic required new strategies for socio-political communication to handle such a crisis. Using the case of India, which was one of the worst-hit countries and is also the world’s largest democracy, this research explicates how political leaders responded to the COVID-19 crisis on Twitter during the first wave as it was the first time such a crisis occurred. Theoretical frameworks of discursive leadership and situational crisis communication theory have been used to analyze interactions based on the usage patterns, the content of communication, the extent of usage in relation to the severity of the crisis, and the possible role of leaders’ position along with the status of their political party. The sample consisted of tweets posted by six prominent political leaders in India across the four consecutive lockdown periods from 25th March to 31st May 2020. A total of 4,158 tweets were scrapped and after filtering for retweets, the final dataset consisted of 2,809 original tweets. Exploratory data analysis, sentiment analysis, and content analysis were conducted. It was found that the tweets had an overall positive sentiment, an important crisis management strategy. Four main themes emerged: crisis management information, strengthening followers’ resilience and trust, reputation management, and leaders’ proactiveness. By focusing on such discursive aspects of crisis management, the study comprehensively highlights how political interactions on twitter integrated with politics and governance to handle COVID-19 in India. The study has implications for the fields of digital media interaction, political communication, public relations, and crisis leadership.

## Introduction

Due to the tremendous increase in computers and internet accessibility along with smartphones, human-media interactions are growing as a major arena for computer-mediated political communication. Political leaders have seized this space for direct communication with their followers since it’s easy to use, wide-ranging, and creates a perception of being more accessible and authentic (Parmelee and Bichard, 2012). In times of crisis, the need for such communication by leaders is magnified. This paper focuses on the usage of the microblogging platform Twitter by some key national political leaders in India during the first wave of COVID-19 pandemic. It raises two important questions; first, what was the content of the communication that was used by the Indian political leaders during the crisis, and second, was there a variation in the content of communication, the extent of usage, and usage pattern of the microblogging platform in relation to the position/role of the leaders in the country.

Twitter provides a space for brief, purposive, and unmediated political communication (Parmelee and Bichard, 2012). The landscape of communication, thereby, has changed to a shorter and a more unmediated mode that promotes direct and informal communication between the leaders and the followers. India ranks third with 18.8 million user accounts on Twitter (Statista Research Department, 2021) suggesting a deep interaction with a large number of people. Narendra Modi, the current Prime Minister of India, is the most followed elected world leader on twitter with 69.6 million followers (India Today, 2021). Research has shown that Twitter has become a source of information and news for many users (Hughes et al., 2012). With the progression of the spread of COVID-19 virus and the changing rules and guidelines to combat it, Twitter was deemed as an authentic source of latest updates and advice from the relevant actors (Haman, 2020). Political communication during the COVID-19 pandemic called for urgent, clear, and efficient sense-making of the crisis. The language used by the leader plays a critical role in framing a chaotic and ambiguous crisis (Whittle et al., 2015) such that it could propel the falling public spirits into a collective hope (Burdett, 1999).

Research has focused on crisis-in-the-moment, wherein, the roles and responsibilities of the leaders are taken to be well-defined, and the unit of study is the progression of the crisis itself, that is, how the crisis is unfolding (for example, Fortunato, 2008). However, the COVID-19 pandemic was not momentary but rather an ongoing reality requiring both retrospective and prospective sensemaking of the situation by the leaders. This meaning making is required as a crisis creates an emotionally charged situation wherein certain negative emotions (such as frustration, anxiety, shock, etc.) rise very soon, thus creating a challenge for decision-making (Hutchins and Wang, 2008). Coombs (2015), regards crisis as “the perception of an unpredictable event that threatens important expectancies of stakeholders and can seriously impact an organization’s performance and generate negative outcomes” (p. 3). Thus the “perception” of the crisis is as significant as the severity of the crisis. Crisis demands an integration of various skills on part of the leaders, as they are required to not simply respond to the crisis, but also manage the perception of the crisis at a larger level. Skills such as authenticity (Gigliotti, 2016), strategic thinking (Wang et al., 2021), effective communication (Wang et al., 2021; Kim and Liu, 2012), empathy (McGuire et al., 2020), humbleness (Scardigno et al., 2021) among others determine the perception of the effectiveness of the leader in managing the crisis.

Crisis, therefore, presents the leaders with the opportunity to enhance their public image in the eyes of the members of the society. Forbes reported that the approval ratings for most leaders around the world has risen since the pandemic (Klebnikov, 2020). For example, Narendra Modi saw a rise in the approval ratings from 62% in the beginning of 2020 to 68% in April 2020, the highest approval rating among all leaders across the globe (Klebnikov, 2020). One reason for such surge in the approval ratings is that, a crisis thrusts the individual reality in a state of shock, distress, and confusion thereby leading to an increased faith in the leaders as a collective coping-mechanism (Bligh et al., 2004). In a crisis, leaders are granted with an intensified form of “proxy control” as the followers rely on leaders for interpreting the events thereby bolstering the trust in leaders’ ability to deal with the crisis (Bligh et al., 2004). As elucidated by Bandura (2001), proxy agency is a situation wherein people who have little direct control or expertise in certain social conditions, relieve their psychological stress by evoking a socially-mediated form of agency. Since the leaders have greater power and access to resources, the followers rely on them to achieve the desired goals (Bandura, 2001).

In this study, political communication was grounded in the context of the leader’s role and status of his/her party in the country. During a national crisis, it is expected that the opposition party would also put up a united front in order to support the government in managing the crisis effectively, and at the same time, portray a positive and empathetic image to the public (Ghosh, 2020). Lewis (2020) suggests that communicating constructive criticism regarding the ruling party’s policies and strategies is a much better position for the opposition party than creating chaos and risking the perception of being opportunistic. These notions echo the arguments put forth by research on categorization and framing (Samra-Fredericks, 2003; Fairhurst, 2007; Whittle et al., 2015), proposing that categories provides frames for meaning making and communication. Categories carry a given set of behaviors, expectations, obligations, and perspectives that the incumbent members are expected to perform (Samra-Fredericks, 2003). This study focuses on these discursive aspects of leadership in tackling the crisis via twitter-mediated communications by leaders from the ruling party, the opposition party, and key-crisis management positions. We categorized six leaders in three categories, namely, ruling party leaders, Narendra Modi (Prime minister) and Amit Shah (Home Minister); opposition party leaders, Rahul Gandhi (key member of a prominent opposition party and member of the parliament) and Arvind Kejriwal (president of a prominent opposition party and the chief minister at the capital of India, Delhi); and key-crisis-management leaders, Harsh Vardhan (Health Minister) and Nirmala Sitharaman (Finance Minister).

Communication by the leaders during the crisis is viewed through the discursive lens, to understand how the leaders indulged in meaning making and communication during the COVID-19 crisis with the general public through media interactions. Some researchers have found the approach of discursive leadership to be fruitful in the context of a crisis (see Clifton, 2012; Gigliotti, 2016; Brown, 2019) but none applied it to an ongoing massive crisis that COVID-19 posed. Leadership is emphasized as a discursive phenomenon - an inherent social construction with a complex interplay of leader, follower, and context characteristics (Hamilton and Bean, 2005). Thus, leadership in this respect is understood as a dialectical and discursive process wherein there is a central focus on the role of communication and context (Fairhurst and Connaughton, 2014).

The situational crisis communication theory (SCCT; Coombs, 2007) provides a comprehensive framework to understand effective communication responses during a crisis. It has been applied in organizational settings previously where it has been asserted that the way organizations respond to a crisis depends upon the type of crisis it faces: victims (e.g., natural disasters), accidental (e.g., technical errors, accidents), or intentional (e.g., intentional misconduct). Even though the pandemic does not lend itself to an organizational setup, the COVID-19 crisis falls into the victim category, wherein the political leaders were also the victims of the crisis. Coombs (2007) emphasizes on two obligatory base responses - instructing information and adjusting information, that must be communicated with the victims of the crisis before the crisis managers can address the reputation management needs. These two responses correspond to how to protect oneself physically and how to cope psychologically, respectively. Though these responses are essential as they visualize how the crisis managers enable people to cope with the crisis, there has been barely any focus on them as effective crisis management strategies (Kim and Liu, 2012). The present study aims to explore the understanding of these two base responses. Another departure that the current work undertakes is that it goes beyond the organizational context and extends SCCT to a national-political situation by focusing on how political leaders communicated about the health crisis to the general public.

### Context of the Study: COVID-19 Pandemic in India

In India, the first case of the disease was reported on 30th January 2020. On the same day, the World Health Organization, triggered a health emergency of global concern for COVID-19. On 11th March 2020, WHO officially declared COVID-19 as a global pandemic. More than a week later, on 19th March 2020, the prime minister of India, Narendra Modi made the first official address to the nation on the issue of the pandemic. In his address, he made a clarion call to follow a self-imposed curfew (#JanataCurfew) on 22nd March, which was obeyed quite diligently by the citizens. This was followed by a 21-day government imposed complete lockdown of the country. Overall, the government imposed four lockdown periods that lasted from March to May 2020. The first lockdown was announced on 24th March at 8 pm to be enforced in just 4-h at midnight. Modi drove home the importance of social-distancing, especially in India, where the healthcare infrastructure is ranked 145th among 195 countries (Fullman et al., 2018). Given the short time period after the announcement of the lockdown, panic and fear rose dramatically (Jha, 2020), people were stranded in different parts of the country with no mode of transportation to reach home, and the uncertainty of life and livelihood seemed to grip the country. The first lockdown ended on 14th April. There was a total of 11,485 cases and 396 deaths. The lockdown was then extended till 3rd May as the 2nd phase of the lockdown. In this phase, areas were classified as red, orange, and green zones according to the number of cases, and restrictions were placed accordingly. The total number of cases rose to 42,778 and the number of deaths accumulated to 1,463 by the end of Lockdown 2. The third phase of the lockdown initiated from 4th May and lasted till 17th May with certain relaxations such as personal vehicle movement, opening of liquor shops, etc. being given in green zones. By the end of the third phase, the total number of cases doubled, 95,698 and the total number of deaths rose to 3,024. With such high numbers, the lockdown was again extended till 31st May as a fourth phase. In this phase, the relaxations were increased as the states were given more liberty to handle the red containment zones. Nevertheless, by the end of this phase, the total number of cases went up to 1, 90,649 and the total number of deaths were 5,406. Even with such alarming statistics, the government decided to reopen the economy, and Unlock 1.0 was initiated from 1st June 2020 onward. We specifically focus on these four phases (during the first wave of the pandemic) as it was the first time this highly unprecedented crisis had posed itself and the world’s largest democracy saw an extended lockdown filled with hysteria and panic; thus, imposing a challenge for crisis management.

According to a study by Oxford, on a scale of 1-100 measuring the stringency index of the government responses to the COVID-19 pandemic, India scored a constant 100 from March 25 to April 19, 2020, that later dipped to approximately 76 toward June 2020 (Hale et al., 2020). The same period was ridden with other socioeconomic complexities such as lack of good healthcare infrastructure (Tejaswi, 2020), lack of resources to conduct education online (Sudevan, 2020), condition of the poor, labor migration (Rashid et al., 2020), uncertainties regarding the progress of the virus, and concerns regarding the falling gross domestic product (OECD, 2020), among others. Against this backdrop, the challenge for the leaders was to pull the people together toward the unified goal of fighting the COVID-19 virus. These four lockdown periods posed unprecedented challenges and also provided the leaders an opportunity to handle it via mass communication via twitter. While previous research has discussed how other world leaders like those from G8 have used twitter during the pandemic (Rufai and Bunce, 2020), we are not aware of any study that has taken a closer look at India although it is one of the most complex countries to deal with having strained healthcare facilities, larger number of cases and is the 2nd most populated country of the world housing about 1/5th of the world’s population. We address the following interrelated aspects by examining the usage of twitter by six important political leaders across the four lockdown periods and accordingly pose the following research questions:

RQ1: What was the extent of Twitter usage by the political leaders in India across the four lockdown periods during COVID-19 crisis?

RQ2: What was the content of communication that was used by the Indian political leaders during the crisis?

RQ3: Did severity of the crisis affect the frequency of communication?

RQ4: Will there be a difference in the content and usage of twitter in relation to the position/role of the leaders in the country?

## Data and Method

### Dataset

The data was extracted from timelines of specific users for a specified time period utilizing Twitter APIs via the OAuth 2.0 Bearer Token using Python (Van Rossum and Drake, 2009). The terms and conditions for data usage specified by Twitter were duly adhered and no further ethical approval was required as the tweets retrieved for the study were from the public domain.

Data consisted of tweets posted by 6 political leaders in India (see Table 1) across the four lockdown periods, that is, from 25th March to 31st May 2020. The inclusion criteria for the political leaders were: popularity index on Twitter (number of followers > 1 million), activity on Twitter (number of posts > 100), verified personal account, and position of the leader in the country. The leaders were categorized in three groups: ruling party leaders, Narendra Modi (Prime minister) and Amit Shah (Home Minister); opposition party leaders, Rahul Gandhi (key member of a prominent opposition party and member of the parliament) and Arvind Kejriwal (president of a prominent opposition party and the chief minister at the capital of India, Delhi); and key-crisis-management leaders, Harsh Vardhan (Health Minister) and Nirmala Sitharaman (Finance Minister). The tweets of the leaders were further divided in four periods in accordance with the lockdown phases in India: Lockdown 1.0: 25 Mar - 14 Apr 2020 (21 days); Lockdown 2.0: 15 Apr - 3 May 2020 (19 days); Lockdown 3.0: 4 May 2020 – 17 May 2020 (14 days); and Lockdown 4.0: 18 May 2020 – 31 May 2020 (14 days).

**TABLE 1:** List of political leaders included in the study.

S.no.	Political leader	Party	Position	Personal twitter handle*	Number of followers*	Number of tweets (25th mar-31st may 2020)	Number of tweets retweeted by other users
	**Ruling party leaders**						
1.	Narendra Modi	Bhartiya Janta Party (BJP)	Prime Minister	@narendramodi	57.9M	618	56,42,983
2.	Amit Shah	Bhartiya Janta Party (BJP)	Home Minister	@AmitShah	20.5M	345	23,66,785
	**Opposition party leaders**						
3.	Arvind Kejriwal	Aam Aadmi Party (AAP)	Chief Minister of Delhi, Capital of India	@ArvindKejriwal	19.2M	482	7,88,700
4.	Rahul Gandhi	Indian National Congress (INC)	Member of Parliament	@RahulGandhi	14.4M	122	14,53,184
	**Key-Crisis-Management leaders**						
5.	Harsh Vardhan	Bhartiya Janta Party (BJP)	Minister of Health and Family Welfare	@drharshvardhan	2.3M	2141	9,63,300
6.	Nirmala Sitharaman	Bhartiya Janta Party (BJP)	Minister of Finance	@nsitharaman	3.3M	450	11,17,530

*Data Source: www.twitter.com as of 1st June 2020.

Initially, 4158 tweets were extracted for all the 6 leaders using python’s Tweepy package and then filtered by removing retweets^1^ so as to retrieve only the original tweets posted by the leaders. Tweets (*n* = 4158) were composed predominantly in the English language (55.51%), followed by Hindi (44.27%), and other languages (0.19%; Gujarati, Marathi, Tamil). The final dataset included 2,809 original tweets (data available upon request from the corresponding author). All the tweets which were not in English (mainly in Hindi) were translated into English using python’s translate package, Python API for Google translate. A random subset of tweets was reviewed to check the accuracy of the translation, and the semantic meaning of the tweets was found to be preserved, leading to accept the translated dataset.

### Text Pre-processing

A tweet is composed of many words, punctuations, URLs, special characters, etc. Before conducting analysis, the text data must be cleaned wherein irrelevant and noisy parts of the text are removed. The basic data cleaning included removal of URLs, hashtags, emoticons, punctuations, symbols, and numbers and all text was transformed to lowercase. The text was tokenized into word-tokens and the stopwords^2^ were removed using the NLTK library in Python. The clean dataset of word-tokens was then used to create word clouds, however, for sentiment analysis the word-tokens were again joined into sentences.

### Analysis

To explore the research questions three types of analysis were conducted, exploratory data analysis, sentiment analysis, and content analysis.

#### Exploratory Data Analysis

Exploratory Data Analysis is the most common Natural Language Processing (NLP) technique. It helps to make sure that the text data has been cleaned thoroughly and therefore is the first type of analysis conducted before moving onto more advanced NLP techniques. Word clouds were created using word cloud library in Python. The visual representation gives a clear perspective about what the political leaders were tweeting about and how they differ in terms of the top words (most commonly used words) used at a glance (data available upon request from the corresponding author).

#### Sentiment Analysis

Sentiment analysis was conducted to understand the polarity of the tweets, that is, how positive, negative, or neutral is a tweet. This analysis helps us to give meaningful insights into the quality of the sentiments (view/opinion in terms of polarity) expressed by the leaders in their tweets. The polarity of the tweets was determined by using the TextBlob package in Python, that provides rule-based sentiment scores ranging from −1 to 1 based on the words in each tweet. Here, each tweet was assigned a single sentiment score, where a negative score denotes a negative sentiment, a positive score denotes a positive sentiment, and 0 denotes a neutral sentiment. Text blob finds all the words and phrases that it can assign polarity to, and then takes the average of all these polarities, hence giving a single polarity score for each tweet. For sentiment analysis, Text blob is considered to be the most accurate for NLP amongst other available libraries (Loria, 2021; Shahul, 2021).

#### Content Analysis

The content analysis was based on the theoretical assumptions of naturalistic inquiry (Lincoln and Guba, 1985) and was conducted in accordance with the steps delineated by Braun and Clarke (2006). After familiarization with the data, the tweets of all the leaders were assigned preliminary codes by a human coder. In this phase, the authors devised the coding scheme by integrating categories commonly identified in previous research with new categories emerging from the data (Bligh et al., 2004; Cho et al., 2013; Rufai and Bunce, 2020; Wang et al., 2021). The 22 initial codes were then organized into 9 themes by examining the relationships within the codes. These relationships are based on the similarity of latent meanings/concepts of the various initial codes. For example, initial codes that reflected the expression of emotions such as hope, empathy, etc., were clubbed under one theme. These themes were further reviewed and refined, where certain themes were reworked upon, some others that did not seem meaningful to the data were dropped, and some new themes that seemed to fit better were created and re-coded. Finally, after sufficient fine-tuning, a coding frame with four main themes was reached. Clear descriptions for each identified message type were produced (see Table 2).

**TABLE 2:** Content analysis themes with descriptions.

S.No.	Themes
1.	**Crisis management information**
	i) Precautionary Measures
	Tweets that describe the precautions and preventive strategies that the individuals can follow to minimize the spread of infection and protect themselves. Example, “social distancing,” “wear a mask,” “wash your hands.”
	ii) Orders
	Tweets regarding executive orders or announcements issued by the government bodies, such as lockdown dates and guidelines, closures of non-essential businesses, schools, services etc., openings of health care facilities, testing camps etc., travelling restrictions, markings of containment zones, etc.
	iii) Situational information
	Tweets that denote the associated risks of not following the guidelines issued by health agencies and also awareness about the nature of the COVID-19 virus. For example, number of cases, recovery rate, spread of virus in particular areas, plasma therapy, how the virus spreads, the need to stay at home, etc.
	iv) Resource provision
	The relief measures taken by the state government, central government, or specific organizations/individuals, to alleviate the impact of the pandemic. Example, arrangements for shelter, ration, cash, hospital beds, financial packages/funds, etc.
	v) Volunteer/donation
	Tweets denoting the need for donations and volunteers.
	vi) Other Media Engagements
	Tweets that inform regarding the public briefings, media interactions, press conferences, discussions with experts, etc., conducted in relation to the pandemic by the leader.
	vii) Opinion and commentary
	Tweets denoting ideas, critical comments, or general opinions in the context of the pandemic, measures being taken, and other pandemic related news.
2.	**Strengthening followers’ resilience and trust**
	i) Empathy
	Tweets denoting an acknowledgment of the pain, hardships, and difficulties being endured by people due to the pandemic.
	ii) Morale-Boosting
	Tweets that seek to boost morale or raise the spirits, and generate a feeling of hope and trust that with the ongoing efforts the pandemic will be overcome.
	iii) Follower’s Worth
	Tweets that denote gratefulness, appreciation, and have positive affirmations for specific individuals or groups. Example, appreciation for the heroic efforts of frontline workers, use of terms such as “corona warriors,” etc.
	iv) Collective Focus
	Tweets that refer to collective unity and the need to fight the pandemic together.
3.	**Reputation management**
	i) constructive criticism/blame
	Tweets that give constructive criticisms or blame toward the actions of the opposition party/govt. agency
	ii) Self-Bolstering
	Tweets that praise own party actions, or information that shows the efforts/actions of their own party in a positive light.
4.	**Leader proactiveness**
	Tweets that describe a course of action that is being taken, can be taken, or will be taken in a certain way.

Two post-graduate students were recruited to annotate the entire dataset and were trained on the coding scheme identified above. In the training, the coding frame was discussed at length, and practice sessions were conducted, wherein the author and the coders together coded the tweets. Each tweet was coded into a single category. In cases of overlap, the majority of the tweet content determined the classification. The inter-rater reliability was calculated in three waves using Cohen’s Kappa (Cohen, 1960). The first-wave reliability, calculated on 5% of the data (*n* = 145), was 0.568 falling in the weak category (McHugh, 2012). The discrepancy was discussed and the coding scheme was further clarified. Next, 20% of the data was coded (*n* = 562) and the reliability estimate improved to 0.676. The inter-rater reliability was 0.852 for the entire dataset which is considered sufficiently strong and acceptable (McHugh, 2012).

## Results

### Usage of Twitter Handles by the Leaders

In terms of number of followers, Narendra Modi (Prime Minister) ranked the highest (57.9 Million) whereas Harsh Vardhan (Health Minister) ranked the lowest (2.3 Million). Modi also had the highest number of retweets (56,42,983), that is, the number of times Modi’s tweets were retweeted by other users, while Arvind Kejriwal (Chief Minister of Delhi) had the lowest number of retweets (7,88,700; see Table 1). Retweets help us understand media interactions and might not just express interest, but also trust in the message and the originator, along with likely agreement with the content (Metaxas et al., 2015).

Of the 2,809 original tweets posted by the leaders from 25th March to 31st May 2020, about 81% (*n* = 2272) of the tweets were related to the COVID-19 crisis. The number of tweets posted by all six leaders differed significantly across the four lockdown periods (χ^2^(1,3) = 188.32, *p* < 0.0001). For all leaders, the frequency of tweets decreased as the lockdown phases progressed. The highest number of original tweets (n = 1611) were posted by the health minister, Harsh Vardhan, while both Rahul Gandhi (Opposition Party leader) and Nirmala Sitharaman (Finance Minister), posted the least number of original tweets (*n* = 115).

### Sentiments Expressed by the Leaders

**FIGURE 1:**
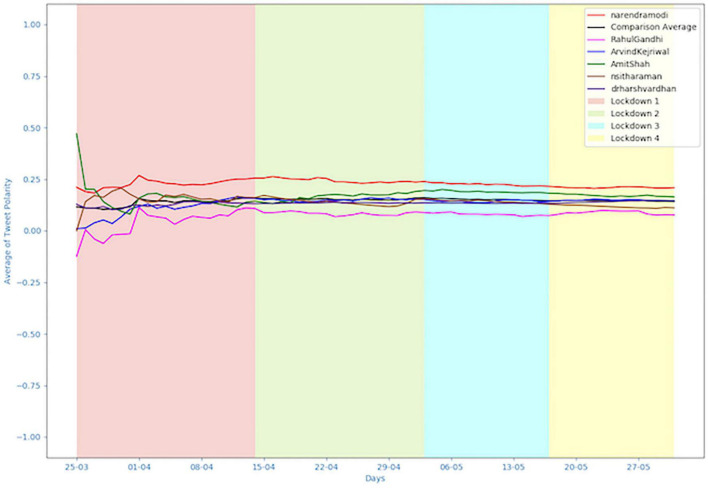
Average Polarity of Tweets posted by the leaders across the four lockdown periods. The graph depicts the average polarities per day (average of sentiment scores of all tweets posted on a particular day by a particular leader) for all the six leaders across the four lockdown periods.

Using the Kruskal-Wallis *H* test, a significant difference was found between the sentiments of the tweets posted by the leaders over a period of 62 days, that is, the four lockdown periods (*H*(5) = 34.351, *p* = 2.027e-06). Kruskal-Wallis *H* test was used as it is a non-parametric test used to test differences between two or more groups on continuous variable (sentiment scores). This test was used as the assumptions of normality required for one-way ANOVA were not met. Further, pairwise comparisons were conducted using Wilcoxon Rank Sum test, and significant differences were found between Arvind Kejriwal and Nirmala Sitharaman (*p* = 0.026), Rahul Gandhi and Harsh Vardhan (*p* = 0.012), Nirmala Sitharaman and Harsh Vardhan (*p* = 0.000), and Narendra Modi was significantly different from all the other 5 leaders (*p*-values ranged from 0.000 to 0.025). As shown in Figure 1, the average polarities of the tweets posted by the leaders all lie above the neutral value. There is little variation in the average polarities of all the leaders across the four lockdown periods. No significant differences were found in the sentiments of the tweets posted over the four lockdown periods as well as for the interaction between leaders and lockdown periods. The volatility of the tweets, that is, the variance of the sentiments of the tweets posted per day by a particular leader measured over a period of 62 days was also calculated. Here, Rahul Gandhi had the highest volatility (0.072) while Harsh Vardhan had lowest volatility (0.003) in tweets (see Table 3). This shows that the sentiments of the tweets posted per day by Rahul Gandhi fluctuated the most while those of Harsh Vardhan fluctuated the least. To see how the sentiments of the tweets fluctuated per day for the six leaders (see Supplementary Figure 6).

**TABLE 3:** Sentiment polarity of tweets by the leaders (in %).

Sentiment polarity	Narendra Modi	Amit Shah	Arvind Kejriwal	Rahul Gandhi	Harsh Vardhan	Nirmala Sitharaman
Positive	62.8	58.3	56.1	53.9	64.2	60
Neutral	27.2	30.5	32.6	14.8	22.6	27.8
Negative	10	11.3	11.3	31.3	13.2	12.2
Volatility	0.022	0.045	0.025	0.072	0.003	0.035

*The values reflect the% of positive, negative, and neutral tweets posted by each leader. Example, Narendra Modi posted 62.8% positive sentiment tweets, 27.2% neutral sentiment tweets, and 10% negative sentiment tweets overall. The volatility of the tweets is the variance of the sentiments of the tweets posted per day by a particular leader measured over a period of 62 days (four lockdown periods).*

### Content Analysis

To answer the question of what is communicated by the leaders in the tweets and whether the content of the communication differs across leaders and the lockdown periods (with rising severity of the crisis), content analysis was conducted wherein four main themes emerged (Table 2). Using the Chi-square test, it was found that the three groups, that is, ruling party leaders, opposition party leaders, and key-crisis management leaders differed significantly in terms of the content of their communication (χ^2^(2,4) = 167.76, *p* < 0.0001) (see Supplementary Table 4). These differences are further qualitatively discussed below.

#### Crisis-Management Information

The leaders communicated about the precautions to be undertaken by the public, the executive orders issued, and the relief measures taken by the government, the need for volunteers and donations, public briefings, and related opinions or comments on the issues arising due to the pandemic. Of the total 2,809 tweets, crisis-management informational tweets formed the majority (30.9%), wherein Harsh Vardhan (Health Minister) was found to make most such tweets (36.8%), followed closely by Arvind Kejriwal (Chief Minister of Delhi; 33.5%) and Amit Shah (Home Minister; 31.8%; Table 4). However, on a closer look, most tweets by Amit Shah were about resource provision. Surprisingly, Modi (Prime Minister) tweeted the least number of informational messages overall but did communicate the most about precautionary measures. Examples of such messages are available upon request from the corresponding author.

**TABLE 4:** Content analysis frequency percentages of the tweets by the leaders (*n* = 2809).

S.no.	Codes	Narendra Modi	Amit Shah	Arvind Kejriwal	Rahul Gandhi	Harsh Vardhan	Nirmala Sitharaman	Total
1.	**Crisis management information**	**13.0%**	**31.8%**	**33.5%**	**28.8%**	**36.8%**	**20.8%%**	**30.9%**
	Precautionary Measures	5.5%	0.6%	2.0%	0.0%	6.6%	3.5%	5.1%
	Orders	0.4%	0.6%	5.0%	0.0%	4.8%	2.6%	3.5%
	Situational information	0.2%	0.0%	9.0%	0.9%	11.0%	0.0%	7.3%
	Resource provision	1.2%	24.9%	11.6%	0.9%	10.7%	7.8%	9.5%
	Volunteer/donation	2.0%	3.4%	0.0%	0.9%	0.4%	2.6%	0.9%
	Other Media Engagements	3.7%	2.3%	5.6%	14.8%	2.9%	2.6%	3.8%
	Opinion and commentary	0.0%	0.0%	0.3%	11.3%	0.4%	1.7%	0.8%
2.	**Strengthening followers’ resilience and trust**	**50.8%**	**21.4%**	**35.9%**	**18.2%**	**20.5%**	**38.3%**	**28.2%**
	Empathy	1.0%	2.8%	2.3%	8.7%	1.7%	0.9%	2.0%
	Morale-Boosting	16.5%	4.5%	10.3%	1.7%	8.8%	12.2%	9.9%
	Follower’s Worth	30.2%	9.0%	19.3%	1.7%	7.6%	21.7%	13.2%
	Collective Focus	3.1%	5.1%	4.0%	6.1%	2.4%	3.5%	3.1%
3.	**Reputation Management**	**1.4%**	**10.2%**	**5.6%**	**14.8%**	**7.6%**	**11.3%**	**6.9%**
	Constructive criticism/blame	0.0%	0.6%	0.3%	11.3%	0.1%	2.6%	0.7%
	Self-Bolstering	1.4%	9.6%	5.3%	3.5%	7.5%	8.7%	6.2%
4.	**Leader Proactiveness**	**15.5%**	**1.1%**	**10.6%**	**8.7%**	**18.0%**	**7.8%**	**14.9%**
5.	**Non-COVID Related Tweets**	**19.2%**	**35.6%**	**14.3%**	**29.6%**	**17.3%**	**21.7%**	**19.1%**
	**Total**	100%	100%	100%	100%	100%	100%	100%

*The values in bold reflect the frequency percentages of tweets posted by each leader for each main theme.*

Previous research on government responses to crisis on social media has found information dissemination to be the most frequent type of message (Heverin and Zach, 2010; Cho et al., 2013; Wukich, 2016; Rufai and Bunce, 2020; Wang et al., 2021). However, few studies delve into what aspects of the crisis are communicated in these information-related messages. For example, the study by Heverin and Zach (2010) made a distinction between information-related and opinion-related messages on Twitter, and Cho et al. (2013) further expanded their typology to include a new distinction for ‘information based on personal experiences.’ Wang et al. (2021) generated 16 categories, based on the typology created by Wukich (2016) for emergency response managers, to examine the type of information disseminated by global, national, and state level health agencies in response to COVID-19 pandemic on Twitter. In this study, only 7 of the 16 categories were found to be relevant in understanding the nuances of the tweets posted by the leaders. All these categories clustered together to form the theme of crisis-management information (Table 2).

#### Strengthening Followers’ Resilience and Trust

Tweets by the leaders expressed empathy, gratefulness, and sought to boost the followers’ morale as well as their worth by praising their efforts and showing trust in their ability to deal with the crisis. Further, the leaders also tried to communicate the need to fight the pandemic collectively thereby creating a shared perception of the crisis. Such messages aimed to strengthen the coping mechanisms of the followers by acknowledging their hardships, appreciating their efforts, and communicating that they are not alone in the crisis. Bligh et al. (2004) argued that leaders use more charismatic language in the wake of a crisis as the followers’ individual reality is thrust into a state of shock, distress, and confusion. Rufai and Bunce (2020) also found that apart from informational and political messages, the G7 world leaders tweeted morale-boosting messages in response to the COVID-19 pandemic. Previous research have marked the presence of emotion-related messages in crisis communication on Twitter (Heverin and Zach, 2010; Kim and Liu, 2012; Cho et al., 2013).

Modi surpassed all the other leaders as 50.8% of his total tweets aimed to strengthen the followers’ resilience and trust (see Table 4). Nirmala Sitharaman (38.3%) and Arvind Kejriwal (35.9%) are close; however, Rahul Gandhi (18.2%) ranked the lowest. Overall, this theme contributed to 28.2% of the total tweets made by the leaders. Examples of such tweets are available upon request from the corresponding author.

#### Reputation Management

Another salient theme that emerged in the analysis was of reputation management wherein the leaders aimed to boost their reputation in the public eye by either communicating their own party actions in a positive light or by criticizing/blaming the other party.

Both Nirmala Sitharaman and Amit Shah posted tweets with high praise of Modi and the actions being taken by the ruling party. Even the tweets that majorly communicated crisis-management information were framed by praising Modi or their own party. For example, Shah (2020) tweeted, “Modi government understands the issues and problems faced by the MSMEs, amidst COVID-19 pandemic. To help them in these stressed times, GoI will facilitate provision of Rs. 20,000 crore as subordinate debt, which will benefit more than 2 lakh MSMEs. #AatmaNirbharBharatAbhiyan.” Even though such tweets are coded under crisis-management information, the reflection of reputation management is worth noting. Interestingly, Modi himself has zero tweets with criticism or blame and very few that seek to boost his own reputation. Similarly, Arvind Kejriwal has very few reputation management tweets. Rahul Gandhi is high on reputation management but his tweets majorly tilt towards blame and criticism for the actions of the ruling party (see Table 4). Another interesting finding is that, both Arvind Kejriwal and Rahul Gandhi have praised the efforts of the ruling government and the need for collaborative action by going beyond the party lines. Though such tweets were quite few in number, there were no such references to cooperation made by the ruling party leaders. Of the total tweets, only 6.9% comprised reputation management.

Work on crisis-communication in the organizational context is replete with research that underline the importance of reputation management to minimize the harm or negative impact of the crisis (Kiambi and Shafer, 2016; Cheng, 2018) and on strategic reputation management employed by public and government organizations when dealing with crisis (Kim and Liu, 2012; Olsson, 2014); which is also echoed in this data. Additionally, praising efforts of ruling party could also be an effective communication strategy by the opposition leaders as outlined before.

#### Leader Proactiveness

Leader preparedness to deal with the crisis in terms of the actions that are being taken or will be taken in the near future was another theme that emerged. Demarcating the tangibility of actions was quite difficult and therefore any references to action, however, proximal or distant in future were coded as leader proactiveness. Of the total tweets, 14.9% comprised of leader proactiveness. Harsh Vardhan, Modi, and Kejriwal frequently posted such tweets as compared to the other leaders. Amit Shah has barely any tweets that refer to leader proactiveness (see Table 4).

## Discussion

Crisis management is thought to be about quick actions, policy-making, and managing technical aspects of the crisis. However, under the COVID-19 health crisis, the challenge for the leaders was to persuade the public to follow new social norms and alter their behaviors. This study emphasizes the rhetorical space provided by Twitter for unmediated communication between the leaders and the followers. The question guiding this research was, how the political leaders in India responded to the crisis on Twitter. Taken together, the findings suggest that the communication by the leaders on Twitter was predominantly around crisis-related messages (around 81%) over the four lockdown periods. Despite the chaos and uncertainty, the messages posted by the leaders had an overall positive sentiment. This positive tilt found in the sentiment suggests that the tweets posted by the leaders were less reflective of the panic and distress caused by the COVID-19 pandemic. Sentiment analysis helps us to understand how (i.e., in what tone) the messages were framed. The extent of twitter usage, however, differed significantly both in terms of the volume and sentiment of tweets among the six political leaders. The analysis gives insight into the strategic choice of leaders to use a more positive frame thereby encouraging their followers that the crisis can be overcome.

### Content of Communication During the Crisis

The rhetoric by the leaders primarily focused on not just information dissemination but also on strengthening the resilience and trust of the public to restore the hope that together the crisis shall be overcome. The findings of the study refocus attention on the two base responses identified as precursors to the application of situational crisis communication theory (Coombs, 2007). The “instructing” and “adjusting” information responses are often either dropped entirely or barely garner a cursory nod by the researchers in preference for the “reputation management” response by the organizations (e.g., Olsson, 2014; Cheng, 2018; Guerber et al., 2020; see also Cheng, 2018). Coombs (2007) writes, “crisis managers must begin their efforts by using communication to address the physical and psychological concerns of the victims” (p. 165). He firmly states that these two responses are obligatory before the crisis managers can address the impression management needs. However, there is a dearth of literature that attempts to further clarify as to what constitutes instructing and adjusting information.

It was found that the leaders’ communication was not limited to precaution or protection-related messages but was rather nuanced. Various aspects of crisis-management information such as precautionary measures, orders and guidelines, nature of the virus, recovery rates, number of cases, availability of hospital facilities and treatments etc., formed a major part of the tweets by the leaders (around 31%). In a similar vein, Kim and Liu (2012) articulated the instructing information response as, messages that provide basic information related to crisis, address primary needs of the public, and specifies the organization’s preparation for the crisis. Previous research on crisis also outlines this desire for information by the public from the leaders to make sense of the crisis (Cho et al., 2013; Spence et al., 2015; Gigliotti, 2016; McGuire et al., 2020). Grint (2010) argues that the leaders have to communicate a persuasive picture of the crisis to the followers even if they themselves are uncertain of the course of action to be undertaken. Therefore, the leaders play a central role in disseminating crisis-management information that helps the public to interpret the threatening and disruptive events in a quick and impactful manner.

It is noteworthy that the tweets denoting the theme of “strengthening follower’s resilience and trust” were almost on par with the informational tweets (28.2%). This study supports Coombs’ assertion that the crisis managers have a responsibility to address the psychological stress and anxiety caused by the uncertainty in a crisis. Kim and Liu (2012) described adjusting information as messages that express sympathy/compassion for the victims and that denote the corrective actions being taken by the organization to deal with the crisis. In this study, however, it was found that the leaders were not just expressing empathy but more predominantly were tweeting messages to boost the followers’ morale (10%), self-worth (13%), and collective unity (3%). These results echo the principles of servant leadership wherein there is a focus on acknowledging the followers’ needs and emotions, establishing the support of community, building trust between the leader and the followers, and calling for actions in common interest (Greenleaf, 1977; Laub, 2004; Matteson and Irving, 2006). Interestingly, both Narendra Modi and Arvind Kejriwal have previously been dubbed as “servant leaders” (Majumdar, 2015). In a crisis, this care-based approach is meaningful as it directs attention to the sufferings and hardships of the people and asserts community welfare as a critical concern (Oliver, 2006; McGuire et al., 2020).

In COVID-19, the need for such coordinated action between the government, public, and other agencies, was indeed high. The sudden imposition of lockdown and various strict measures required a high level of trust between the leaders and the followers. Due to the lack of a foreseeable future, new orders, guidelines, and measures were being announced without any prior notice. Given this, the leaders were also actively communicating about their actions (about 15%), thereby making the followers privy to the process of how they are managing the crisis. Leader proactiveness has been marked as a delectable competency in times of a crisis (Stoller, 2020) and inspires hope and trust in leaders’ effectiveness. Even though the crisis-management literature is abounding with studies focusing on reputation management (Kim and Liu, 2012; Olsson, 2014; Kiambi and Shafer, 2016; Cheng, 2018), in the present study, quite a diminished focus was found on this aspect by the leaders overall (around 7%). However, it is worth noting that it was quite elusive to tap this aspect as even the informational or trust invoking messages were often framed in a manner that bolsters the reputation of the leaders or their parties. No matter the grave and alarming nature of the COVID-19 crisis, reputation-management remains an important theme of the communication by the leaders.

### Influence of Severity of Crisis

The number of tweets went down as the severity of the crisis increased. The parameters for charting the severity of crisis were number of cases, number of deaths, and increasing economic difficulties due to the lockdown. It was found that the initial shock and absurdness of the crisis warranted more communication from the leaders. Around 35% of the total tweets were posted in lockdown 1 and this number substantially dropped to 17% by Lockdown 4.

**FIGURE 2:**
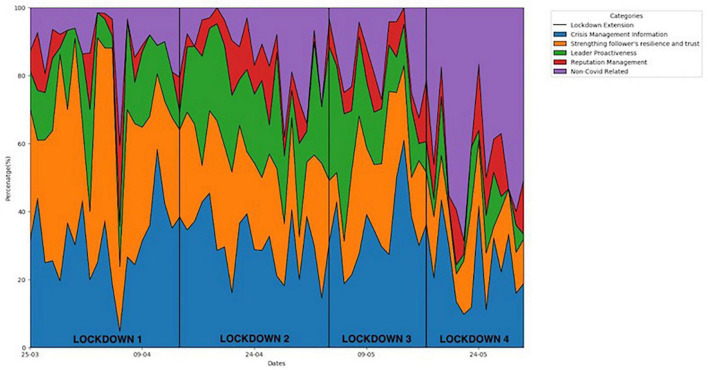
Changing data percentages of different message types over four lockdown periods.

Figure 2, shows that during Lockdown 1, strengthening followers’ resilience and trust took precedence over all other types of messages. Crisis-management information tweets were more stable in comparison with only a slight drop in lockdown 4. Recent studies also report that mobilizing collective effort, enabling coping, and providing crucial information are imperative for building trust in the leadership during crisis (Haslam and Reicher, 2016; Wilson, 2020). Interestingly, even though the number of cases were rising at an alarming level, the greatest chunk of tweets during lockdown 4 comprised non-COVID related tweets (47%). The results bring forth the enduring human tendency of seeking normalization even in the vicissitudes of a crisis. Both the need and the focus of the leaders’ tweets shifted away from COVID-19 as the severity of the crisis increased.

### Tweeting From the Party Window

**FIGURE 3:**
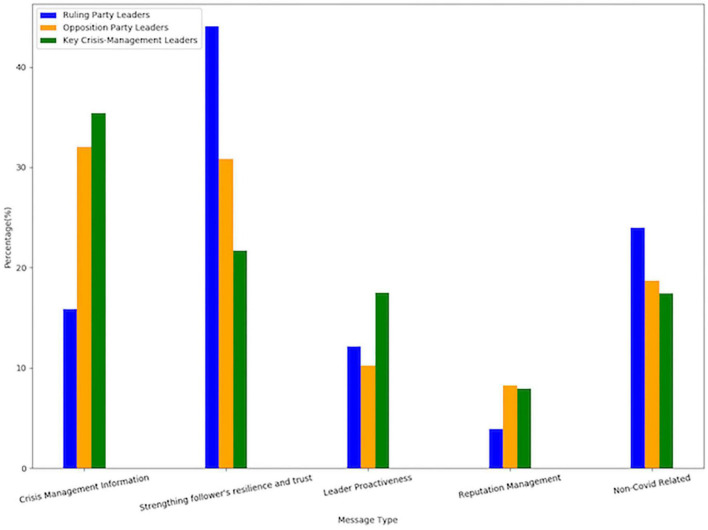
Percentages of different message types with respect to the position/status of the leaders.

In this study, leaders’ communication was viewed from the perspective of their position in the country and the status of their party. The six leaders comprised three groups, ruling party leaders (Modi and Shah), opposition party leaders (Arvind Kejriwal and Rahul Gandhi), and key crisis-management leaders (Harsh Vardhan and Sitharaman). It was found that the content of the communication differs with respect to the leaders’ position and status of their party (see Figure 3). The key crisis-management leaders posted the highest number of crisis informational tweets (35.7%) as well as tweets denoting leader proactiveness (17.3%), whereas the ruling party leaders stood out in terms of posting tweets that seeks to boost the resilience and trust of the followers’ in overcoming the crisis (43%). Interestingly, the opposition party leaders (8.2%) were found to be in stiff competition with the key crisis-management leaders (7.9%) in terms of reputation management, even though the overall communication for this category was low.

Coombs (2007) posited that crisis creates an opportunity to shape/change peoples’ perception of the organization through communication. From Table 3, it can be seen that the ruling party leaders and key-crisis-management leaders posted more positive sentiment tweets as compared to the opposition party leaders, wherein Rahul Gandhi posted the highest percentage of negative sentiment tweets. The crisis response strategies of these individual leaders gain greater clarity when seen through the lens of their positions in the country. Modi’s crisis response strategy of posting the positive tweets that aim to bolster the morale and trust of the followers lines up well with his role of prime minister and the leader of the ruling party. Even though he has the highest number of followers and therefore a greater reach, he posted the lowest number of informational tweets. More than how the crisis is being managed or how to protect oneself from the virus, his focus was on enabling the psychological coping of the people. Since Sitharaman and Harsh Vardhan are also members of the ruling party, they together with Amit Shah use the self-bolstering strategy of reputation management by highlighting how well the ruling government is managing the crisis under the leadership of Modi. For example, Shah (2020) tweeted “Truth is self evident! Entire world is praising PM @narendramodi, the way he is handling COVID-19 global pandemic, taking care of Indians and helping the world community in such challenging times. Every Indian is feeling safe and trusts his leadership.” Modi does not concern himself with reputation management in his tweets, but the trumpeting is well-managed by the rest of the ruling party leaders. As health and finance ministers, Harsh Vardhan and Nirmala Sitharaman, duly held their roles by communicating the varied aspects of information regarding the crisis, activities being undertaken, and encouraging trust and resilience in people on Twitter.

For the opposition, COVID-19 crisis has been dubbed as a “new page,” since the sole focus was now on healthcare infrastructure and the status of the economy (Vij, 2020). Rahul Gandhi’s tweets had the highest negative sentiment and mostly denoted opinions, comments, and constructive criticism. The discussion on the political landscape in India is beyond the scope of this paper, but it must be noted that Gandhi’s approach in response to the COVID-19 crisis has been considered as a more mature “constructive opposition” as compared to his previous political strategies (Vij, 2020). In his tweets, he brings out the dismaying reality of the crisis, provides alternative strategies, and holds the ruling party leaders accountable for their actions. Both Rahul Gandhi and Arvind Kejriwal also declared their support for the ruling party leaders in their tweets, an element missing from the tweets of the latter. Arvind Kejriwal finds himself midway between the opposition and the ruling party. His role of chief minister of the national capital gives him a more active vantage point than Rahul Gandhi but limits it to the state rather than national level. Nevertheless, he is one of the most followed opposition party leaders on Twitter in India (Socialbakers, 2020). His tweets were quite balanced in terms of spreading crisis information as well as strengthening peoples’ morale and resilience.

There are few studies that contextualize twitter communication using the frames of the leaders’ roles and position. One example is a study by William Ie (2020), that focused on how the political leaders’ tweets reflect their leadership roles and helps in the construction of a more unmediated leader-follower relationship. In this study, two contextual variables are taken into account, severity of the crisis and position/role of the leader. There is a need to look at more variables, such as, economic indexes, socio-political history, among others, to understand how leaders respond to a crisis.

## Limitations

The study also has several limitations. First, the data was only limited to Twitter and other digital and traditional media platforms were not included. Future studies can undertake various other sources through which leaders communicate, such as public briefings, interviews, Facebook posts, Prime Minister’s *Mann ki baat* (radio program), etc. Second, the data and analysis were textual in nature whereas other factors such as tone, facial expressions, body language, etc., also impact on how the leaders’ messages are interpreted. Third, how the responses of the leaders interact with the macro-level social, economic, and political factors was not included in the analysis. Fourth, there was no comparison sample used, that is, how the leaders communicated before the pandemic. Fifth, the study was based only on six Indian political leaders, future studies can include other political leaders active on Twitter and draw more nuanced comparisons both nationally and internationally. Sixth, the crisis response of the leaders is studied under COVID-19 health crisis, and the results may not be generalizable to other types of crisis. And lastly, we did not analyze interaction of followers with the leaders on twitter but rather focused on a one-sided communication by the leaders only.

## Conclusion

This paper examined the crisis communication of six key political leaders in India in response to the COVID-19 pandemic on Twitter during the four lockdown periods in the first wave. The findings offer three main contributions to the fields of crisis leadership, public relations, communications, and digital media interaction.

First, the study extends the application of SCCT to a public health crisis and adds greater theoretical clarity to the base crisis responses proposed by Coombs (2007) by providing empirical evidence. Very few studies focus on how the content of communication changes in relation to severity of crisis. The study highlights the importance of tweets enabling coping in the first phases of the crisis, the imminent role of informational tweets throughout the crisis, and the tendency of human nature to shift to other areas/topics as the crisis extends with no regard to the severity level. A key finding looks at how crisis communication emanates from the roles/position of the leader and reiterates the need to contextualize analysis of leader communication. Second, the study focuses on discursive aspects of crisis management on Twitter, at a time when new media technologies are being highly integrated with politics and governance. Twitter is emerging as a powerful platform that shapes the perception of approachability and authenticity (McGuire et al., 2020). Empirical evidence on how the political leaders were using this medium and what they were communicating in real time will be key in understanding the long-term impact of pandemic on the perception of leaders’ effectiveness. More importantly, Twitter has become a primary source for information consumption and the findings of the study can help the leaders become more cognizant of the messages they are posting and fully tap its potential. Third, this is the first study as far as we are aware of that highlights human-media communication as a window to strategic political communication which offers an analysis of leadership in India.

The findings provide a ground to understand how the tough reality of the pandemic was communicated by the leaders over four lockdown periods from the standpoints of their role/position in the country.

## Data Availability Statement

The original contributions presented in the study are included in the article/Supplementary Material, further inquiries can be directed to the corresponding author/s.

## Author Contributions

NJ, SM, and PS formulated the research idea and questions. PM and NJ did data extraction and analysis. NJ conducted content analysis and wrote the draft. SM and PS edited the draft. All authors contributed to the article and approved the submitted version.

## Conflict of Interest

The authors declare that the research was conducted in the absence of any commercial or financial relationships that could be construed as a potential conflict of interest.

## Publisher’s Note

All claims expressed in this article are solely those of the authors and do not necessarily represent those of their affiliated organizations, or those of the publisher, the editors and the reviewers. Any product that may be evaluated in this article, or claim that may be made by its manufacturer, is not guaranteed or endorsed by the publisher.

## Footnotes

1 Retweets are tweets that are posted by a particular user and are then shared or re-posted by another user. Only those retweets were excluded wherein no additional comment/remark was made by the leaders.

2 Stopwords are those words in the English language that are commonly used but hold little meaning in text analysis (such as “the,” “a,” “an,” “between,” etc.). Some other words unique to the data (such as ‘amp’, ‘ji’, ‘shree’, ‘rs’, ‘shri’, ‘rt’, ‘mr’, ‘smt’) were also added to the list of stopwords. It must be noted here that the data cleaning process took multiple iterations as certain errors or certain insights cropped up when conducting analysis. Text-preprocessing and analysis are often circular even though they are reported in a linear fashion (Zhao, 2018).

## References

[B1] Bandura A. (2001). Social cognitive theory: an agentic perspective. *Annu. Rev. Psychol.* 52 1–26. 10.1146/annurev.psych.52.1.1 11148297

[B2] Bligh M. C. Kohles J. C. Meindl J. R. (2004). Charisma under crisis: presidential leadership, rhetoric, and media responses before and after the September 11th terrorist attacks. *Leadersh. Q.* 15 211–239. 10.1016/j.leaqua.2004.02.005

[B3] Braun V. Clarke V. (2006). Using thematic analysis in psychology. *Qual. Res. Psychol.* 3 77–101. 10.1191/1478088706qp063oa 32100154

[B4] Brown C. P. (2019). *Discursive Leadership: Exploring the “Black Box” Challenge in Transcultural Leadership Studies Ph. D. thesis.* San Diego, CA: University of San Diego.

[B5] Burdett J. O. (1999). Leadership in change and the wisdom of a gentleman. *Particip. Emp. Int. J.* 7 5–14. 10.1108/14634449910262474

[B6] Cheng Y. (2018). How social media is changing crisis communication strategies: evidence from the updated literature. *J. Conting. Crisis Manag.* 26 58–68. 10.1111/1468-5973.12130

[B7] Cho S. E. Jung K. Park H. W. (2013). Social media use during Japan’s 2011 earthquake: how twitter transforms the locus of crisis communication. *Media Int. Aust.* 149 28–40. 10.1177/1329878X1314900105

[B8] Clifton J. (2012). A discursive approach to leadership: doing assessments and managing organizational meanings. *J. Bus. Commun.* 49 148–168. 10.1177/0021943612437762

[B9] Cohen J. (1960). A coefficient of agreement for nominal scales. *Educ. Psychol. Meas.* 20 37–46. 10.1177/001316446002000104

[B10] Coombs W. T. (2007). Protecting organization reputations during a crisis: the development and application of situational crisis communication theory. *Corp. Reput. Rev.* 10 163–176. 10.1057/palgrave.crr.1550049

[B11] Coombs W. T. (2015). *Ongoing Crisis Communication: Planning, Managing and Responding*, 4th Edn. Thousand Oaks, CA: Sage publication.

[B12] Fairhurst G. T. (2007). *Discursive Leadership.* Thousand Oaks, CA: Sage publication.

[B13] Fairhurst G. T. Connaughton S. L. (2014). Leadership: a communicative perspective. *Leadership* 10 7–35. 10.1177/1742715013509396

[B14] Fortunato J. A. (2008). Restoring a reputation: the duke university lacrosse scandal. *Public Relat. Rev.* 34 116–123. 10.1016/J.PUBREV.2008.03.006

[B15] Fullman N. Yearwood J. Abay S. M. Abbafati C. Abd-Allah F. Abdela J. (2018). Measuring performance on the healthcare access and quality index for 195 countries and territories and selected subnational locations: a systematic analysis from the global burden of disease study 2016. *Lancet* 391 2236–2271. 29893224 10.1016/S0140-6736(18)30994-2 PMC5986687

[B16] Ghosh A. (2020). *COVID19: Reinforcing the Role of Political Opposition in India.* New Delhi: Observer Research Foundation.

[B17] Gigliotti R. A. (2016). Leader as performer; leader as human: a discursive and retrospective construction of crisis leadership. *Atlant. J. Commun.* 24 185–200. 10.1080/15456870.2016.1208660

[B18] Greenleaf R. K. (1977). *Servant Leadership A Journey into the Nature of Legitimate Power and Greatness.* New York, NY: Paulist Press.

[B19] Grint K. (2010). The cuckoo clock syndrome: addicted to command, allergic to leadership. *Eur. Manag. J.* 28 306–313. 10.1016/j.emj.2010.05.002

[B20] Guerber A. J. Anand V. Ellstrand A. E. Waller M. A. Reychav I. (2020). Extending the situational crisis communication theory: the impact of linguistic style and culture. *Corp. Reput. Rev.* 23 106–127. 10.1057/s41299-019-00081-1

[B21] Hale T. Webster S. Petherick A. Phillips T. Kira B. (2020). *Oxford COVID-19 Government Response Tracker.* Available online at: https://www.bsg.ox.ac.uk/research/research-projects/oxford-covid-19-government-response-tracker (accessed September 30, 2020). 10.1038/s41562-021-01079-8 33686204

[B22] Haman M. (2020). The use of twitter by state leaders and its impact on the public during the COVID-19 pandemic. *Heliyon* 6:e05540. 10.1016/J.HELIYON.2020.E05540 33294685 PMC7695954

[B23] Hamilton F. Bean C. J. (2005). The importance of context, beliefs and values in leadership development. *Bus. Ethics Eur. Rev.* 14 336–347. 10.1111/J.1467-8608.2005.00415.X

[B24] Haslam S. A. Reicher S. D. (2016). Rethinking the psychology of leadership: from personal identity to social identity. *Daedalus* 145 21–34. 10.1162/daed_a_00394

[B25] Heverin T. Zach L. (2010). “Microblogging for crisis communication: examination of twitter use in response to a 2009 violent crisis in the Seattle-Tacoma, Washington area,” in *Proceedings of the 7 th International ISCRAM Conference*, Seattle, WA.

[B26] Hughes D. J. Rowe M. Batey M. Lee A. (2012). A tale of two sites: twitter vs. facebook and the personality predictors of social media usage. *Comput. Hum. Behav.* 28 561–569. 10.1016/J.CHB.2011.11.001

[B27] Hutchins H. Wang J. (2008). Organizational crisis management: unexplored territory in HRD. *Adv. Dev. Hum. Resourc.* 10 310–330. 10.1177/1523422308316183

[B28] India Today (2021). *PM Modi Now Most Followed Active Politician on Twitter After Suspension of Trump’s Account.* Noida: India Today.

[B29] Jha D. N. (2020). *Coronavirus Fear Goes Viral: Why You Shouldn’t Panic. The Times of India.* Available online at: http://timesofindia.indiatimes.com/articleshow/74466528.cms?utm_source=contentofinterest&utm_medium=text&utm_campaign=cppst (accessed March 4, 2020)

[B30] Kiambi D. M. Shafer A. (2016). Corporate crisis communication: examining the interplay of reputation and crisis response strategies. *Mass Commun. Soc.* 19 127–148. 10.1080/15205436.2015.1066013

[B31] Kim S. Liu B. F. (2012). Are all crises opportunities? a comparison of how corporate and government organizations responded to the 2009 flu pandemic. *J. Public Relat. Res.* 24 69–85. 10.1080/1062726X.2012.626136

[B32] Klebnikov S. (2020). *Most World Leaders See Approval Ratings Surge amid Coronavirus. Not Trump. Forbes.* Available online at: https://www.forbes.com/sites/sergeiklebnikov/2020/04/18/most-world-leaders-see-approval-ratings-surge-amid-coronavirus-not-trump/#78311b40e5a0 (accessed April 18, 2020).

[B33] Laub J. (2004). *Defining Servant Leadership: A Recommended Typology for Servant Leadership Studies.* Virginia Beach, VA: Regent University.

[B34] Lewis H. (2020). *The Essential Role of Opposition During a Pandemic–The Atlantic.* Available online at: https://www.theatlantic.com/international/archive/2020/05/coronavirus-pandemic-opposition-politics-starmer-johnson/611839/ (accessed May 20, 2020).

[B35] Lincoln Y. S. Guba E. G. (1985). *Naturalistic Inquiry.* Beverly Hills, CA: Sage Publications.

[B36] Loria (2021). *Textblob-Research and Compare. Technology Evolution Centers.* Available online at: https://www3.technologyevaluation.com/solutions/53875/textblob (accesed November 13, 2021).

[B37] Majumdar S. (2015). *The New Servant Leader. Business Standard.* Available online at: https://www.business-standard.com/article/opinion/the-new-servant-leader-business-standard-opinion-115021200723_1.html (accessed 11 November 2020).

[B38] Matteson J. A. Irving J. A. (2006). Servant versus self-sacrificial leadership: a behavioral comparison of two follow-oriented leadership theories. *Int. J. Leadersh. Stud.* 2 36–51.

[B39] McGuire D. Cunningham J. Reynolds K. Matthews-smith G. (2020). Beating the virus: an examination of the crisis communication approach taken by New Zealand Prime Minister Jacinda Ardern during the Covid-19 pandemic. *Hum. Resour. Dev. Int.* 23 361–379. 10.1080/13678868.2020.1779543

[B40] McHugh M. L. (2012). Interrater reliability: the kappa statistic. *Biochem. Med.* 22 276–282. 10.11613/bm.2012.031 PMC3900052 23092060

[B41] Metaxas P. Mustafaraj E. Wong K. Zeng L. O’Keefe M. Finn S. (2015). “What do retweets indicate? Results from user survey and meta-review of research,” in *Proceedings of the International Association for the Advancement of Artificial Intelligence Conference on Web and Social Media*, (Menlo Park, CA: PKP Publishing Services Network).

[B42] OECD (2020). *In OECD economic outlook.* Paris: Organisation for Economic Co-operation and Development.

[B43] Oliver C. R. (2006). Catastrophe’s impact on leaders’ caring and justice: changes in moral reasoning orientation. *Int. J. Leadersh. Stud.* 1 80–98.

[B44] Olsson E. K. (2014). Crisis communication in public organisations: dimensions of crisis communication revisited. *J. Conting. Crisis Manag.* 22 113–125. 10.1111/1468-5973.12047

[B45] Parmelee J. H. Bichard S. L. (2012). *Politics and the Twitter Revolution: How Tweets Influence the Relationship between Political Leaders and the public.* Minneapolis, MN: Fortress Press.

[B46] Rashid O. Anand J. Mahale A. (2020). *India Coronavirus Lockdown Migrant Workers and their Long March to Uncertainty. The Hindu.* Available online at: https://www.thehindu.com/news/national/india-coronavirus-lockdown-migrant-workers-and-their-long-march-to-uncertainty/article31251952.ece (accessed September 12, 2020).

[B47] Rufai S. R. Bunce C. (2020). World leaders’ usage of Twitter in response to the COVID-19 pandemic: a content analysis. *J. Public Health* 42 510–516. 10.1093/pubmed/fdaa049 32309854 PMC7188178

[B48] Samra-Fredericks D. (2003). Strategizing as lived experience and strategists’ everyday efforts to shape strategic direction. *J. Manag. Stud.* 40 141–174.

[B49] Scardigno R. Papapicco C. Luccarelli V. Zagaria A. E. Mininni G. D’Errico F. (2021). The humble charisma of a white-dressed man in a desert place: pope francis’ communicative style in the Covid-19 Pandemic. *Front. Psychol.* 12:3586. 10.3389/fpsyg.2021.683259 34539488 PMC8440828

[B50] Shah A. (2020). *No Title.* Available online at: https://twitter.com/AmitShah (accessed 13 May 2020).

[B51] Shahul E. S. (2021). *Sentiment Analysis in Python: TextBlob vs Vader Sentiment vs Flair vs Building It From Scratch”. Neptuneblog.* Available online at: https://neptune.ai/blog/sentiment-analysis-python-textblob-vs-vader-vs-flair (accessed November 12, 2021).

[B52] Socialbakers (2020). *Most popular Twitter accounts in India- Politics.* Available online at: https://www.socialbakers.com/statistics/twitter/profiles/india/society/politics (accessed 12 November 2020).

[B53] Spence P. R. Lachlan K. A. Lin X. Greco M. D. (2015). Variability in Twitter content across the stages of a natural disaster: implications for crisis communication. *Commun. Q.* 63 171–186. 10.1080/01463373.2015.1012219

[B54] Statista Research Department (2021). *Leading Countries Based on Number of Twitter Users as of April 2021.* Available online at: https://www.statista.com/statistics/242606/number-of-active-twitter-users-in-selected-countries/ (accessed 29 June, 2021).

[B55] Stoller J. K. (2020). Reflections on leadership in the time of COVID-19. *BMJ Leader* 4 77–79. 10.1136/leader-2020-000244

[B56] Sudevan P. (2020). *Why e-Learning isn’t a Sustainable Solution to the COVID-19 Education Crisis in India.* Available online at: https://www.thehindu.com/sci-tech/technology/why-elearning-is-not-a-sustainable-solution-to-the-covid19-education-crisis-in-india/article31560007.ece (accessed September 22, 2020).

[B57] Tejaswi M. (2020). *Healthcare Infrastructure in Smaller Towns, Villages Inadequate to Handle COVID-19: Survey.* Available online at: https://www.thehindu.com/news/national/healthcare-infrastructure-in-smaller-towns-villages-inadequate-to-handle-covid-19-survey/article32391185.ece (accessed 19 August, 2020).

[B58] Van Rossum G. Drake F. L. (2009). *Python 3 Reference Manual.* Scotts Valley, CA: CreateSpace.

[B59] Vij S. (2020). *How Covid-19 is Changing Indian Politics.* Available online at: https://theprint.in/opinion/covid-19-changing-indian-politics/391118/ (accessed 12 November 2020).

[B60] Wang Y. Hao H. Platt L. S. (2021). Examining risk and crisis communications of government agencies and stakeholders during early-stages of COVID-19 on Twitter. *Comput. Hum. Behav.* 114:106568. 10.1016/j.chb.2020.106568 32982038 PMC7508681

[B61] Whittle A. Housley W. Gilchrist A. Mueller F. Lenney P. (2015). Category predication work, discursive leadership and strategic sensemaking. *Hum. Relat.* 68 377–407. 10.1177/0018726714528253

[B62] William Ie K. (2020). Tweeting power: the communication of leadership roles on prime ministers’ twitter. *Polit. Govern.* 8 158–170. 10.17645/pag.v8i1.2530

[B63] Wilson S. (2020). Pandemic leadership: lessons from New Zealand’s approach to COVID-19. *Leadersh.* 16 279–293. 10.1177/1742715020929151

[B64] Wukich C. (2016). Government social media messages across disaster phases. *J. Conting. Crisis Manag.* 24 230–243. 10.1111/1468-5973.12119

[B65] Zhao A. (2018). *Natural Language Processing in Python.* Available online at https://www.youtube.com/watch?v=xvqsFTUsOmc (accessed 23 October 2020).

