# Supplementary material for: Twitter Mediated Sociopolitical Communication During the COVID-19 Pandemic Crisis in India
Source: Front Psychol. 2021 Dec 24;12:784907. doi: 10.3389/fpsyg.2021.784907 (PMC8740330; doi:10.3389/fpsyg.2021.784907)
Supplement: Supplementary file 1 [file Data_Sheet_1.pdf]

Supplementary Material

Figure1: Wordcloud for Amit Shah

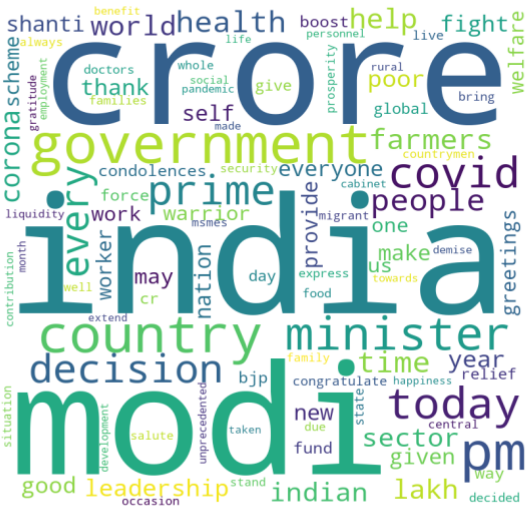

Figure2: Wordcloud for Narendra Modi

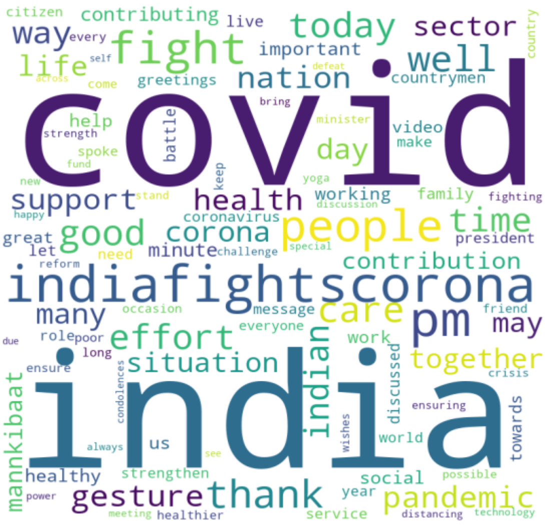

Figure3: Wordcloud for Arvind Kejriwal

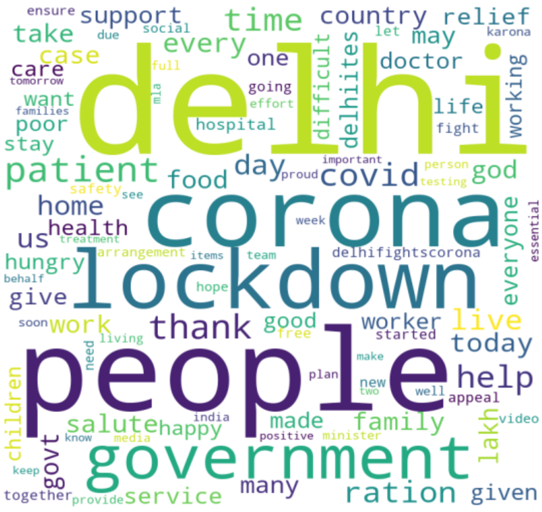

Figure4: Wordcloud for Rahul Gandhi

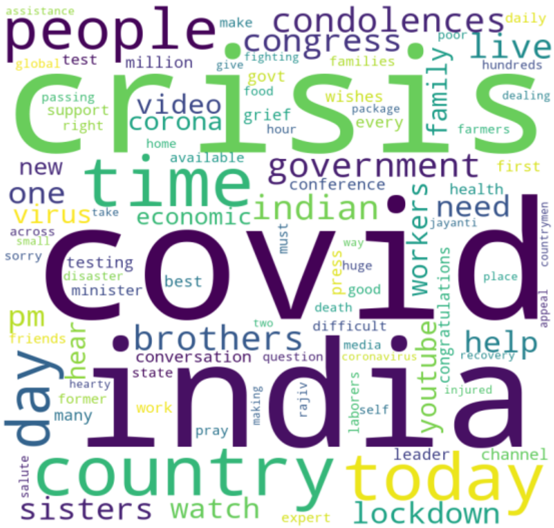

Figure5: Wordcloud for Harsh Vardhan

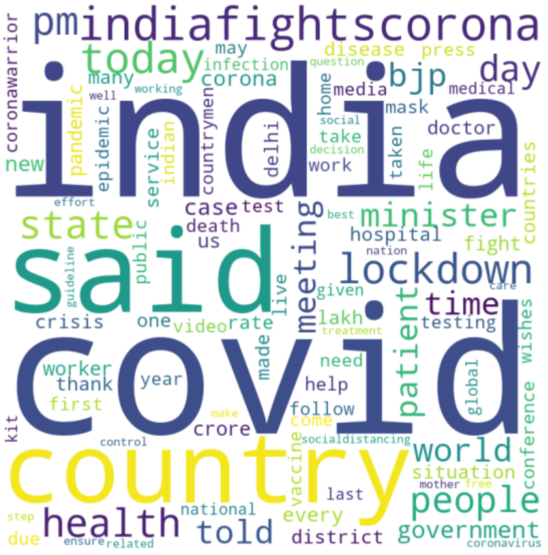

Figure6: Wordcloud for Nirmala Sitharaman

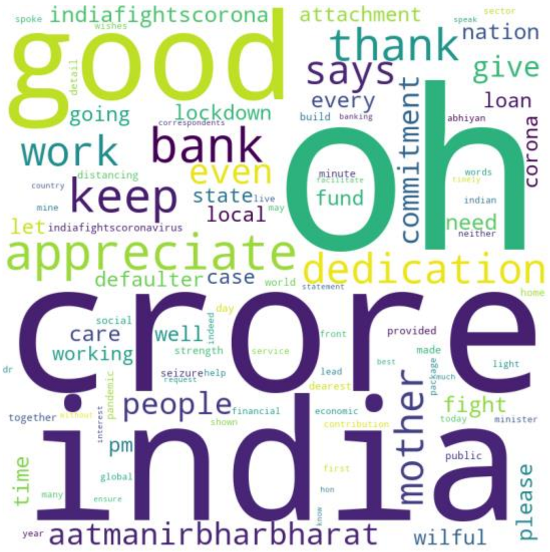

Figure 6: Depicts how the sentiment polarity of tweets posted by the six leaders fluctuates per day

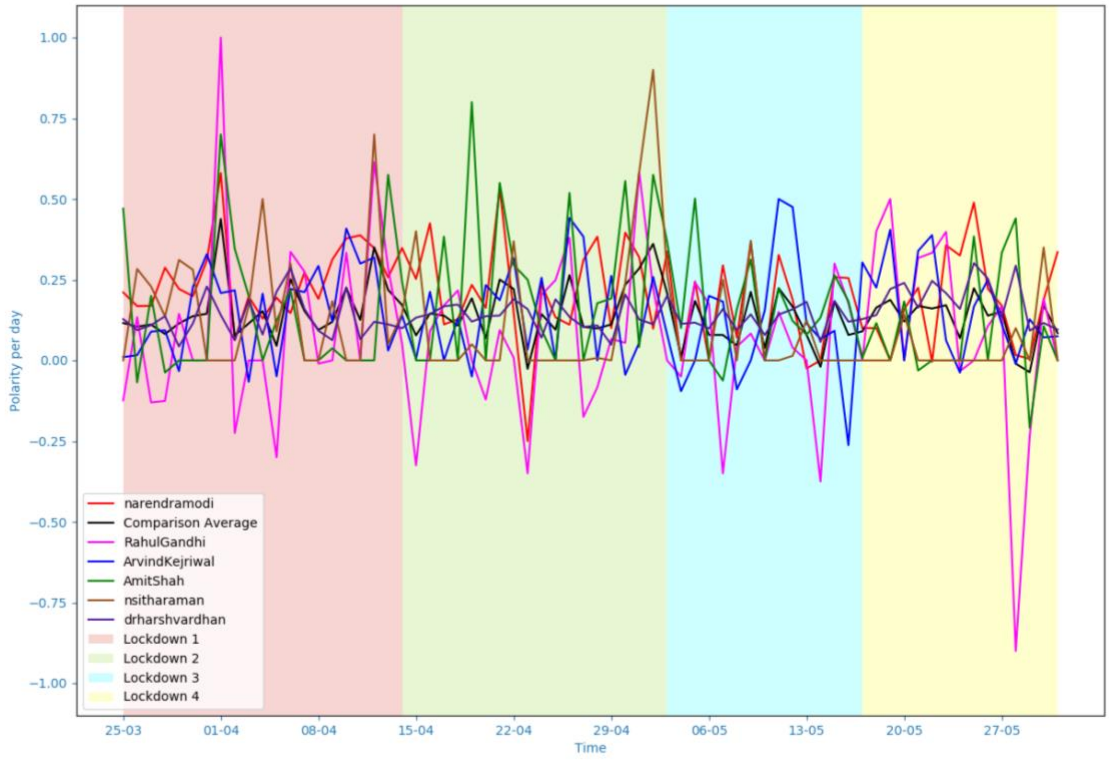

Table 1: Number of original tweets posted by the 6 political leaders across lockdown periods

Lockdown	Narendra Modi	Amit Shah	Arvind Kejriwal	Rahul Gandhi	Harsh Vardhan	Nirmala Sitharaman	Total
**L1**	279	54	150	31	414	55	983
**L2**	106	30	65	34	454	35	724
**L3**	42	62	38	22	428	19	611
**L4**	63	31	48	28	315	6	491
**Total**	**490**	**177**	**301**	**115**	**1611**	**115**	**2809**

Table 2: Lockdown wise content analysis

Themes	L1	L2	L3	L4	Total
**Crisis management information**	32.6%	30.2%	35.2%	23.3%	30.9%
**Strengthening followers' resilience and trust**	38.5%	25.4%	24.9%	15.3%	28.2%
**Reputation Management**	5%	7.6%	7.3%	9.2%	6.9%
**Leader Proactiveness**	12.9%	20.9%	19.0%	5.1%	14.9%
**Non-COVID Related Tweets**	10.9%	15.9%	13.6%	47.3%	19.1%

Table 3: Ruling vs Opposition vs Key crisis managers

S.no.	Codes	Ruling Party Leaders	Opposition Party Leaders	Key Crisis Leaders
1.	**Crisis management information**	**17.9%**	**32.2%**	**35.7%**
	Precautionary Measures	4.2%	1.4%	6.4%
	Orders	0.4%	3.6%	4.6%
	Situational information	0.1%	6.7%	10.3%
	Resource provision	7.5%	8.7%	10.5%
	Volunteer/donation	2.4%	0.2%	0.5%
	Other Media Engagements	3.3%	8.2%	2.9%
	Opinion and commentary	0.0%	3.4%	0.5%
2.	**Strengthening followers' resilience and trust**	**43%**	**31%**	**21.7%**
	Empathy	1.5%	4.1%	1.7%
	Morale-Boosting	13.3%	7.9%	9.0%
	Follower's Worth	24.6%	14.4%	8.5%

	Collective Focus	3.6%	4.6%	2.5%
3.	**Reputation Management**	**3.7%**	**8.2%**	**7.9%**
	Constructive criticism/blame	0.1%	3.4%	0.2%
	Self-Bolstering	3.6%	4.8%	7.6%
4.	**Leader Proactiveness**	**11.7%**	**10.1%**	**17.3%**
5.	**Non-COVID Related Tweets**	**23.5%**	**18.5%**	**17.6%**
	**Total**	**100%**	**100%**	**100%**

Table 4: Contingency table for Chi-square (difference in content of communication across the three groups, i.e., ruling party leaders, opposition party leaders, and key-crisis management leaders

**Themes**	**Ruling Party**	**Opposition Party**	**Key Crisis management**
Crisis management information	120	134	615
Strengthening followers' resilience and trust	287	129	374
Reputation Management	25	34	135
Leader Proactiveness	78	42	299
Non-Covid related	157	77	303
